# Binding property of HIV p24 and Reverse transcriptase by chalcones from Pongamia pinnata seeds

**DOI:** 10.6026/97320630014279

**Published:** 2018-06-30

**Authors:** Manikannan Mathaiyan, Arumugam Suresh, Rangasamy Balamurugan

**Affiliations:** 1 Centre for Drug Discovery and Development, Sathyabama Institute of Science and Technology, Chennai-600 119; 2 Central Research Laboratory, Sri Manakula Vinayagar Medical College & Hospital, Madagadipet, Puducherry- 605107

**Keywords:** HIV, Pongamania pinnata, chalcones, Glabarachalcone, karanijin, P24 protein, Reverese transcriptase

## Abstract

HIV remains a challenging life threatening viral agent for humans despite available anti HIV drugs. The known effective drug named HAART clears the circulating viruses but not the intracellular viruses. Therefore, it is of interest to identify molecules with improved anti-HIV activity from natural plant sources. Hence, we studied the anti-HIV potency of an Indian medicinal plant named Pongamia pinnata. Aqueous extracts were made from leaf, seed and roots of Pongmia pinnata and screened for anti HIV-1 activity using HIV-1 p24 and reverse transcriptase (RT) inhibition assays. Further, the active chalcone derivatives namely, P24 protein and RT enzymes showed promising binding score against Glabarachalcone and Karanijin. Among these extracts, P. pinnta aqueous seed extracts have shown HIV-1 p24 inhibition at 66.9 ± 4.4 percentage. However, RT inhibition assay showed only 36.8%. Hence, the HIV-1 p24 inhibition infers either the prevention of virus entry or inhibits other enzymes and or interferes with virion assembly.

## Background

Currently, there are about 30 anti HIV compounds approved by US FDA for clinical use. This includes the highly active antiretroviral therapy (HAART). HAART is the effective method of choice to treat HIV/AIDS patients. These compendiums of drugs are so powerful to arrest HIV and in majority of the patients it cleared the virus from circulation and keeps the plasma viral load to zero [1]. The problem with this therapy is that the drug needs to be administered continuously and if withdrawn the plasma viral load will reappear.

Anti-HIV drugs also known to cause side effects to the level to which the patient may force to discontinue taking anti-HIV drugs. Hence, there is a need for improved drugs against HIV- 1/AIDS. Globally traditional medicinal system and its derivatives have been used as curative agent of many dreadful diseases. Hence, we used Pongamia pinnata a medicinal plant, traditionally used in India for treating viral skin infections [2]. We have earlier reported the immune modulation property of P. pinnata [3]. Therefore, it is of interest to identify novel inhibitors from P. pinnata against HIV-1/AIDS.

## Methodology

### P. pinnata extracts anti HIV-1 inhibition assays

P.pinnata leaf, root and seed aqueous extracts were prepared at different concentrations and treated with HIV-1clade-C virus. Briefly, 50 μg to 1000μg of different concentrations (50, 100, 200, 400, 800, 1000 μg) of aqueous extracts were used for screen anti HIV-1 inhibition assays. For the anti-HIV testing HIV-1 (Clade C), strain was infected with human PBMCs and TCID 50 was calculated as described in our earlier studies [4] and stored at - 196°C for further use.

For anti-HIV testing, all these extract at different concentrations were pretreated with 15 pg of live virus and incubated for 1 hour at 37°C. After this, virus/extract mixture was added to MT-2 cells (0.3x106 cells) and incubated at 37°C for 2 hours. Cells were washed and re-suspended in 2 % RPMI and further incubated for 5 days at 37°C. Cells treated with Nonoxynol-9, served as positive control, cells treated with distilled water used a s negative cotrol(drug-negative control). After 5 days, the supernatants were collected and tested for HIV-1 gag p24 content by ELISA (Cat. No. XB-1000). Based on the standard curve, HIV-1 p24 concentrations in the treated cultures were calculated.

In addition to HIV-1 p24, inhibitory activity of P. pinnata on HIV RT was also performed. For this, HIV RT assay kit (Roche USA Cat. No.11 468 120 910) was used and the assay was performed as per the manufacturers instruction. 4ng (20μl) of HIV-1- RT in the reaction tube, 20 μl of samples (extracts) or controls was added and this was mixed with 20μl of reaction mixture and incubated for an hour at 37°C. These samples were then transferred to wells of the Micro Plate module; the plate was covered and incubated for 1 hour at 37°C. The plates were then washed with wash buffer for five times. To that 200μl anti- DIG- POD working dilution was added and incubated for one hour at 37°C. The plates were again washed with wash buffer five times. To that 200 μl of ABTS substrate solution was added and incubated at room temperature until a green color develops. The plate was then read at 405 nm in ELISA reader (Biotek, USA). Lysis buffer without RT was used as a negative control and Azidothymidine (AZT) with RT was used as a positive control.

### Phytochemical analysis of P. pinnata seed extract

#### Preliminary Phytochemical Screening

P. pinnata seed extract was subjected to preliminary phytochemical screening of various plant constituents such as alkaloids, flavonoids, Saponins, Carbohydrates, Phenols, Triterpenoids and Glycosides with standard protocol [5].

#### Phytochemical analysis of P. pinnata seed extract by GC-MS

In this experiment aqueous extracts were evaluated for the active compounds, which are possibly associated for the observed bioactivities. P. pinnata seed extracts were subjected for compounds identification by injecting 1μl of extracts into GC-MS (JEOL GC mate) instrument. After running for 40 minutes, major compounds were identified by comparing with standard references [6].

### Molecular Docking of P24 and Reverse transcriptase with chalcones

#### Protein Preparation

The X-ray crystallographic structures of protein target such as P24 HIV-1 (PDB id: 1SJE) and Reverse transcriptase HIV-1 (PDB id: 2JLE) were retrieved from the Protein Data Bank. Water molecules, ligands and other heteroatoms were removed from the protein molecule. Addition of hydrogen atoms to the protein was performed using CHARMm force field. Energy minimization was performed by using conjugate gradient method with an RMS gradient of 0.01kcal/Å mol on Argus lab [7].

#### Molecular Docking

The grid-based molecular docking method is used here using the program tool was used from Argus lab. 4.0.1 ver, which employs CHARMm forcefield. The target is held rigid while the ligands are allowed to be flexible docking. Since the ligands are retrieved drug bank, Canada as per GC MS results. Commercially available antiviral drugs were analyzed with target as a control for docking. Hence it is possible, however, to specify the ligand placement in the active site using a binding site sphere. Then the prepared ligands such as Glabarachalcone, Isopongachromene and Karanijin are docked to the active site using default parameters. The results of the docking enabled the ranking of the docked conformation of the ligands according to their docking score and hydrogen-binding site. Based on standard antiviral drug, chalcones compound were selected as hits for the target protein [7].

#### Analyses and Visualization of the ligand binding sites

The docking poses were ranked according to their docking scores. The scoring function in docking score was used to predict the binding affinity of one ligand to the target molecule. In addition to the structural information, each record includes the docking score reported as negative value, where the higher value indicates a more favorable binding. This enables the energy to be used like a score. This score includes internal ligand strain energy and receptor-ligand interaction energy, and is used to sort the poses of each input ligand. The molecular visualizations of the docked complexes were analyzed using the Argus lab version 4.0.1.

## Result and Discussion

### P24 and Reverse transcriptase suppression activity

**Figure 1:**
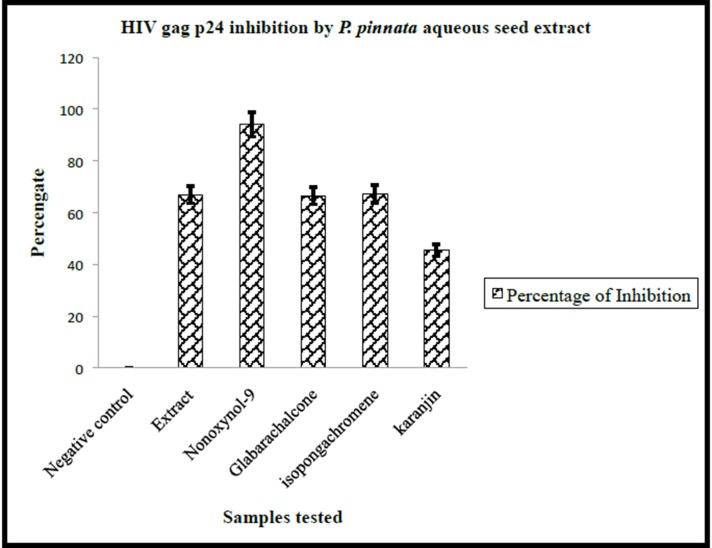
Illustrates the HIV-1 gag p24 inhibition by P. pinnata aqueous seed extracts (100 μg/ml). 0.3x106 MT-2 cells were infected with HIV-1, supernatants collected after 5 days and tested for HIV-1 gag p24 content by ELISA. Neg. cont.=Distilled water treated cultures. Nonoxynol-9 treated cultures (positive control). HIV-1 gag p24 inhibition levels were represented in percentages. All the experiments carried out for three times.

**Figure 2:**
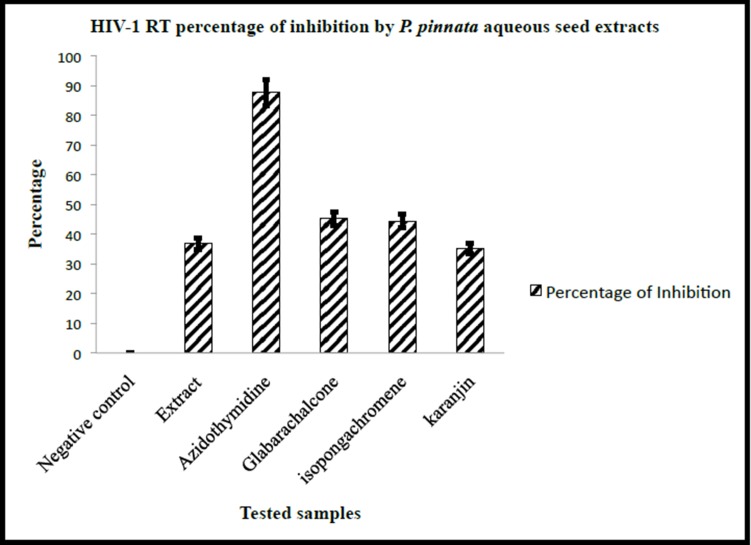
Illustrates the HIV-1 RT inhibition by P. pinnata aqueous seed extracts. 100 μg/ml concentrations of the P. pinnata aqueous seed extracts were treated with HIV-1 RT and reaction mixture. Lysis buffer without RT used as a negative control and Azidothymidine (AZT) with RT used as positive control. HIV-1 RT inhibition was represented in percentages; all the experiments were done at three times.

Of the all extracts screened against HIV-1 p24, significant of inhibition was noticed with seed aqueous extracts at 100 μg/ml in comparison to drug negative control (p < 0.001). As shown in the Figure 1 (data shown for seed aqueous extracts at 100 μg/ml, data not shown for leaf and root extracts and other concentrations) the inhibition ranged between 62.3 percentage to 71.1 % and the mean inhibition was 66.9 ± 4.4. These inhibitions were significantly higher when compared to the drug negative control group, which showed zero percentage inhibition. Though nonoxynol-9 group showed an inhibition higher than 90 percent, the inhibition percentage was found to be significant compared to the drug negative control group. It is important to record that P. pinnata seed extract indeed showed remarkable anti HIV activity. To confirm these findings additional experiments were performed to find out whether this type of inhibition could be found only with p24 alone or the extract could suppress HIV reverse transcriptase enzyme (Figure 2). As shown in the Figure RT inhibition by P. pinnata seed extract was only 36.8 ± 2.4 and these observations suggest that indeed the extract failed to show major inhibitory activity. This observation indicates that HIV p24 inhibition may not be due to RT inhibition pathway.

In our study 2 different assay systems, have been used. One directly tested on the viruses (HIV gag p24 inhibition assay) and the other reverse transcriptase inhibition assay (RT inhibition assay- kit method), which study the inhibitory activity of P. pinnata on one of the most prominent enzyme, reverse transcriptase (indirect assay). In HIV p24 assay it was found that there was a 66.9±4.4-percentage inhibition of HIV virion. However RT inhibition assay showed only 36.8% inhibition suggesting a partial inhibition. This partial RT inhibition while a better percentage of HIV-1 p24 inhibition suggests that the anti HIV activity of P. pinnata could be due to other that RT inhibition. The HIV p24 inhibition could be attributed to P. pinnata prevention at the virus entry level or inhibit other enzymes or interfere with virion assembly.

### Phytochemical analysis of P. pinnata

Initial screening of phytochemical showed the presence of alkaloids, flavonoids, carbohydrates, phenols, steroids, and glycosides. This extract did not contain saponins and triterponoids. GC-MS study of the same extract showed several compounds of which one of the compounds was found to be predominant. There was a chalcone compound known as (E)-1-7(7-hydroxhy-2-2dimethylchromen-6-yl)-3-phenylprop-2-en-1-one (chemical formula C20H18O3, Molecular weight 306.6 kDa) with a peak area of 37.93 (Table 2 - available with authors). Chalcones are key precursors in the synthesis of many biologically important heterocycles such as benzothiazepine, pyrazolines, 1,4-diketones, and flavones. Glabaarachalcone, isopongachromene, and Karanjin were isolated from P. pinnata aqueous seed extracts by standard spectral procedures. This is a heterocyclic compound known to have several antimicrobial activities. Heterocyclic rings are present in several compounds such as vitamin B complex, antibiotics, chlorophyll, haemin, other plant pigments, amino acids and proteins, drugs, dye stuffs, enzymes, the genetic material DNA etc. Chalcones are biosynthetic products of the shikimate pathway, belonging to flavanoid family. These are the precursors of open chain flavonoids and isoflavonoids. Phytochemical analysis showed that our extract perhaps had flavonoids. Thus flavonoid fractions of the extract probably possess (E)-1-7(7-hydroxhy-2-2dimethyl chromen-6-yl)-3- phenylprop-2-en-1-one, which may be responsible for the bioactivity exhibited by P. pinnata [8, 9]. This compound is a chalcone that is commonly found in flavonoids and chalcones are known to exhibit several antimicrobial and anticancer activities. Indeed P. pinnata aqueous seed extract possessed flavonoid fraction. Thus it is predicted that the compound (E)-1-7(7- hydroxhy-2-2dimethyl chromen-6-yl)-3-phenylprop-2-en-1-one that is more abundant in P. pinnata extract could be responsible for the observed antiviral activity.

**Figure 3:**
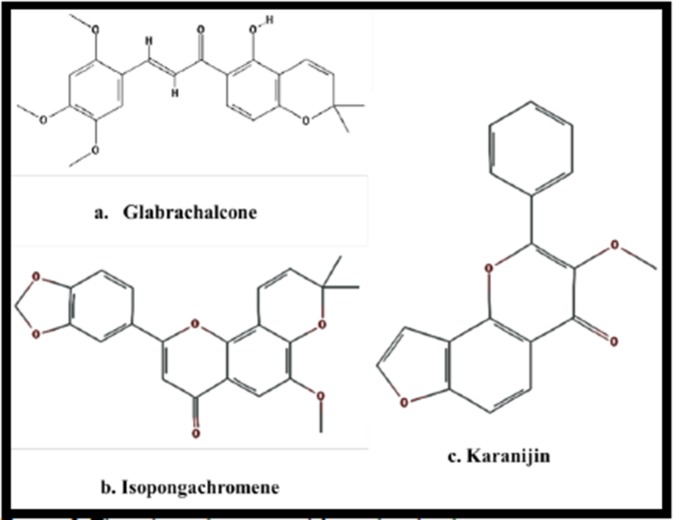
Three ligands retrieved from drugbank.ca.

In our previous study P. pinnata seed extracts showed a strong immune modulatory effect but it also enhanced antiviral immunity. There was observed a relatively higher amount of HIV-1 gag p24 inhibition with P. pinnata. However, similar level of inhibition was not observed with HIV-1 RT suggesting that HIV p24 expression was not RT dependent. Chalcones possess an array of bioactivities and the immune modulant and antiviral activity revealed by this study could be mediated by C20H18O3, which is a potent chalcone. Hence with the chemical structure available, it is possible to chemically synthesize this compound for further characterization. The chemical structure is given in Figure 3, [9].

As per data obtained from GC-MS, Chalcones chemical structure were downloaded from drug bank chalcones considered as a ligand, they docked with P24 protein and reverse transcriptase clearly support the observation of the above two proteins docked with chalcones (Table 1). The three ligands in the docking score in the range of -8 to -11 kcal/mol in contrast to the Reverse transcriptase Glabarachalcone and Karanijin having values of -12 and -9 kcal/mol respectively. Isopongachromeneshows less value of docking score because of its bulky multi ring structures Since Glabarachalcone and Karanijin are showing a higher negative value of docking score comparable to Isopongachrompene and are having the same molecular formula, they would be the likely candidates to be selected for further studies.

In support of the previous analyses and results, Glabarachalcone interacted with Reverse transcriptase by making five H-bonds with a distance <2.9 Å among them, H-bond interaction with 797T, 797T, 877F, 796N and 796N. Similarly, Karanijin interacted with P24 by making three H-bonds; among them H-bond interaction with 573A, 571M, 389H. We were interested to find out the common motifs/residues in P24 and Revertse transcriptase that interact with three ligands. Thus, this analysis strongly supports the earlier observation that Glabarachalcone and Karanijin are the best possible candidates that can be taken up further in search of a drug candidate.

## Conclusion

In this study we found that P. pinnata aqueous seed extracts contained various phytochemicals, especially flavonoids a predominant compound, (E)-1-7(7-hydroxhy-2-2dimethyl chromen-6-yl)-3-phenylprop-2-en-1-one (chemical formula C20H18O3; Molecular weight 306.6 kDa) is a chalcone. P24 protein and RT enzyme for showed promising binding score against Glabarachalcone and Karanijin. The docking results were compared with standard anti-retroviral drug abacavir.

## Figures and Tables

**Table 1:** Docking scores of Chalcone derivative against P24 and Reverse transcriptase targeted protein

	Ligand Name	Hydrogen Bond	Score	Kcal/mol	H bond Binding position	Distance
P24 HIV-1(PDB id: 1SJE)							
Chain B	Glabaarachalcone	3	-9.71349	-10.0272	256T	2.95
					191L	2.99
					191L	2.77
	Isopongachromene	2	-9.71349	-10.0272	241T	2.85
					237D	2.82
	Karanjin	3	-8.4519	-10.0851	215E	2.89
					217S	2.57
					215E	2.89
Reverse transcriptase HIV-1 (PDB id: 2JLE)						
Chain A:	Glabarachalcone	2	-11.118	-11.9834	101K	2.99
					181Y	2.89
	Isopongachromene	3	-8.8474	-9.44609	183Y	2.51
					232Y	2.59
					96H	2.99
	Karanijin	0	-11.65	-12.0191	-	-
	abacavir	3	-7.9704	-8.63354	228L	2.71
					229T	2.89
					228L	2.75

**Figure 4:**
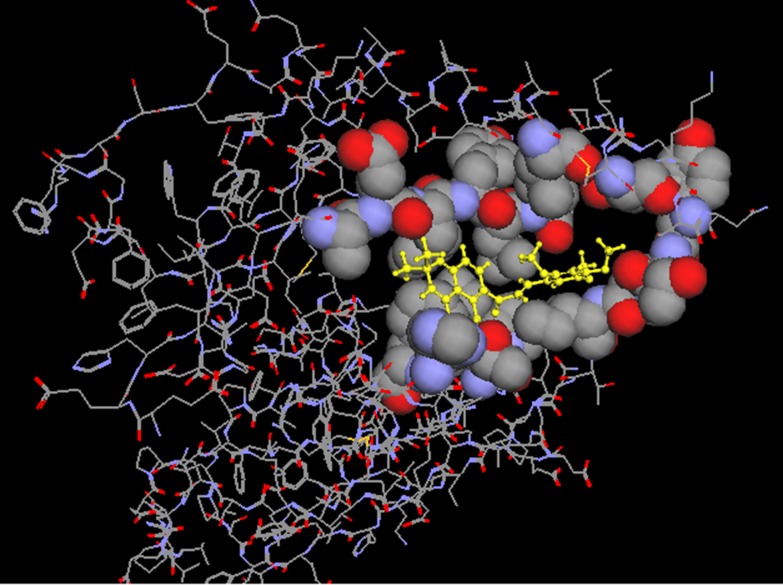
Interacting portion of the Glabarachalcone ligand with HIV-1 P24.

**Figure 5:**
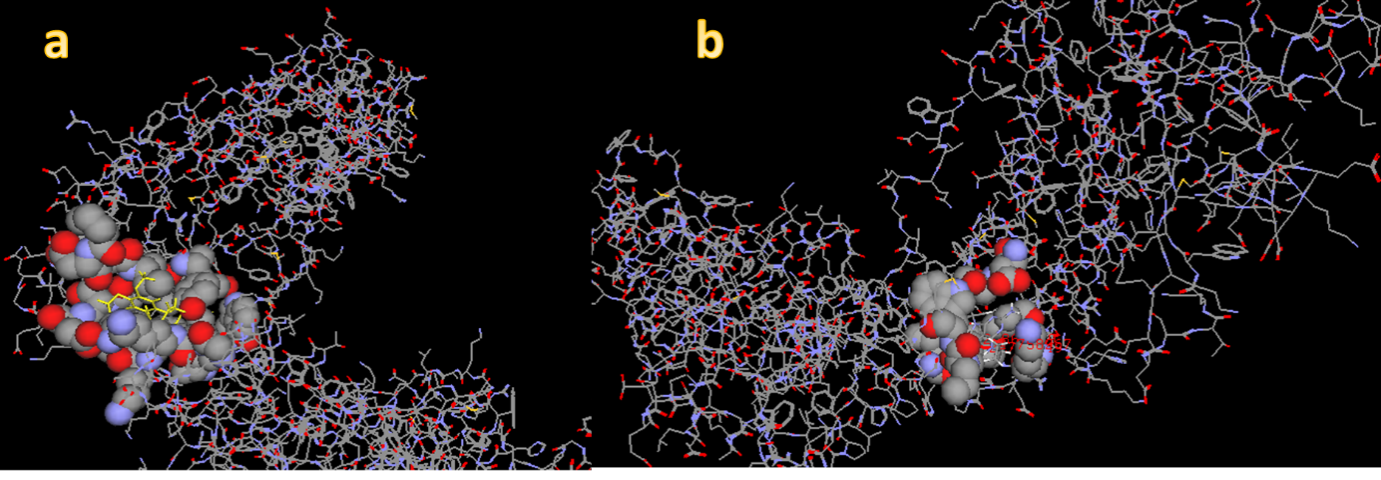
Interacting portion of the ligands with HIV Reverse transcriptase, a) With Glabarachalcone from Pongamania Pinnata; b) with abacavir commercial antiviral drug.
